# Urinary Dickkopf-3 Predicts GFR Loss in the General Population

**DOI:** 10.1016/j.ekir.2025.07.007

**Published:** 2025-07-15

**Authors:** Jon Viljar Norvik, Danilo Fliser, Bjørn Odvar Eriksen, Toralf Melsom

**Affiliations:** 1 Metabolic and Renal Research Group, Institute of Clinical Medicine, UiT The Arctic University of Norway, Tromsø, Norway; 2 Section of Nephrology, Clinic of Medicine, University Hospital of North Norway, Tromsø, Norway; 3 Department of Internal Medicine IV, Nephrology and Hypertension, Saarland University Medical Center, Homburg/Saar, Germany

**Keywords:** biomarker, chronic kidney disease, Dickkopf-3, epidemiology, glomerular filtration rate, uDKK3

## Abstract

**Introduction:**

Chronic kidney disease (CKD) affects > 800 million people globally, with prevalence expected to increase, emphasizing the need for effective early detection and management strategies. Urinary Dickkopf-3 (uDKK3), a profibrotic glycoprotein from renal tubular cells, has been linked to acute kidney injury (AKI) and CKD progression. This study assesses uDKK3 as a biomarker for predicting glomerular filtration rate (GFR) decline in the general population.

**Methods:**

We conducted a prospective cohort study with 1316 participants aged 55 to 69 years from the Renal Iohexol Clearance Survey (RENIS) (2014–2015), undergoing 1 to 3 GFR measurements using iohexol clearance over 5.3 years. uDKK3 levels were normalized to creatinine (Cr), categorizing participants by uDKK3/Cr levels: undetectable, low (detectable < 400 pg/mg), and high (≥ 400 pg/mg). Linear mixed models were used to evaluate the relationship between uDKK3/Cr levels and GFR decline rate. Logistic regression was used to examine the association between uDKK3/Cr groups and accelerated GFR decline, defined as the top 10% steepest declines.

**Results:**

The mean annual GFR decline rate was −1.33 ml/min per 1.73 m^2^. A total of 1112 individuals had undetectable uDKK3/Cr levels, 167 had low levels, and 37 had high levels. The high uDKK3/Cr level group comprised 63% men and had higher systolic and diastolic blood pressure (BP) levels, despite comparable use of antihypertensive medications. Higher baseline uDKK3/Cr levels significantly correlated with faster GFR decline, independent of traditional CKD risk factors. Participants with high uDKK3/Cr levels had an annual GFR reduction 0.63 ml/min per 1.73 m^2^ faster than those with undetectable levels (*P* = 0.049). High uDKK3/Cr levels had an odds ratio of 2.68 for accelerated GFR decline, compared with those with undetectable levels (*P* = 0.03).

**Conclusion:**

Elevated uDKK3 levels were linked to steeper GFR decline, independent of conventional CKD risk factors, confirming uDKK3 as a promising biomarker for early identification of individuals at risk for rapid GFR loss.

## Graphical abstract

**Figure:**
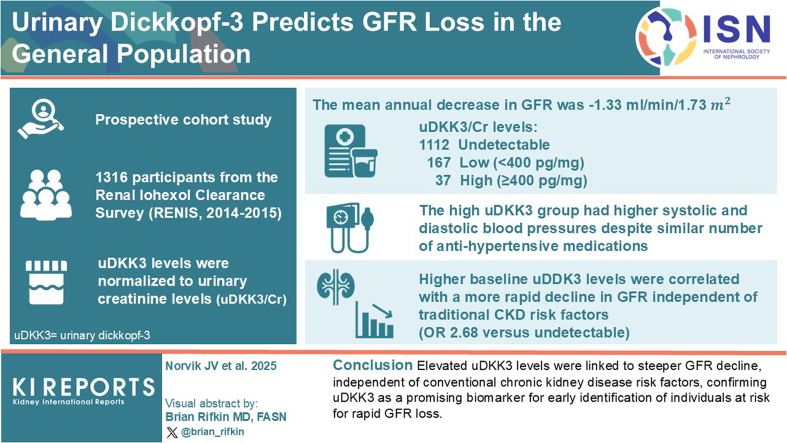

CKD represents a significant global health challenge, affecting > 800 million people worldwide; a prevalence exceeding 10%.^1^ As of 2022, CKD ranks as the 10th leading cause of death globally and it is projected to increase to the sixth leading cause by 2050.^2^ It is currently the 23rd leading cause of disability-adjusted life years and is expected to become the 10th by 2050, with an age-standardized disability-adjusted life year rate increase of 29% over the same period.^2^ This escalating trend underscores the need for effective early detection and management strategies to mitigate the growing impact of CKD on global health.

Early recognition of CKD can limit the progression to end-stage kidney disease and prevent cardiovascular disease by implementation of therapies and counsel on the avoidance of nephrotoxic agents.^3^ However, CKD is difficult to diagnose at its earlier stages in part because of limitations on the most ubiquitous CKD biomarkers, estimated GFR (eGFR) from serum Cr and/or cystatin C and urinary albumin-to-Cr ratio (ACR). Several non-GFR–related factors can influence the eGFR so that it becomes a biased estimate of GFR.^3^ Moreover, in individuals with kidney disease, the GFR may be normal or even elevated, suggesting a maladaptive increase in filtration (hyperfiltration) because of hemodynamic factors.^4^ Furthermore, a significant proportion of patients with CKD exhibit disease progression in the absence of albuminuria, termed the nonproteinuric pathway of CKD progression.^5^

Estimated GFR and ACR are both mainly biomarkers of the glomerulus, indicating glomerular dysfunction or injury.^6^ However, arteriosclerosis, tubular atrophy, and tubulointerstitial fibrosis, which are common features of most CKD etiologies, are not fully captured by eGFR and ACR.^6^^,^^7^ Tubular biomarkers that could supplement eGFR and ACR in detection of CKD at earlier stages will likely improve patient care and have the potential to reduce morbidity and mortality.^3^^,^^6^

Previous research suggests that uDKK3, a profibrotic glycoprotein, is associated with AKI, short-term GFR decline, and progression of CKD.^8^, ^9^, ^10^ DKK3 is produced by stressed renal tubular cells, and uDKK3 is almost exclusively of renal tubular cell origin, making it especially suitable for detecting early tubular injury.^11^, ^12^, ^13^ The studies so far have mostly focused on uDKK3 as biomarker of GFR decline in subjects with existing CKD, employing eGFR from serum levels of Cr. The performance of uDKK3 as a biomarker for measured GFR decline in a general population has not previously been studied.

In the RENIS, we therefore investigated the potential of uDKK3 as a biomarker for a rapid GFR loss in a general population, using GFR measured by iohexol clearance.

## Methods

### Study Population

The RENIS started out as RENIS-T6, a substudy of the sixth Tromsø study. It was conducted in the municipality of Tromsø, located in the Northern Norway. Participants who did not report a history of diabetes, cardiovascular disease, or kidney disease were considered for inclusion in RENIS-T6, which was conducted between 2007 and 2009. The process of inclusion in both Tromsø 6 and RENIS-T6 has been extensively described elsewhere.^14^

**Figure 1:**
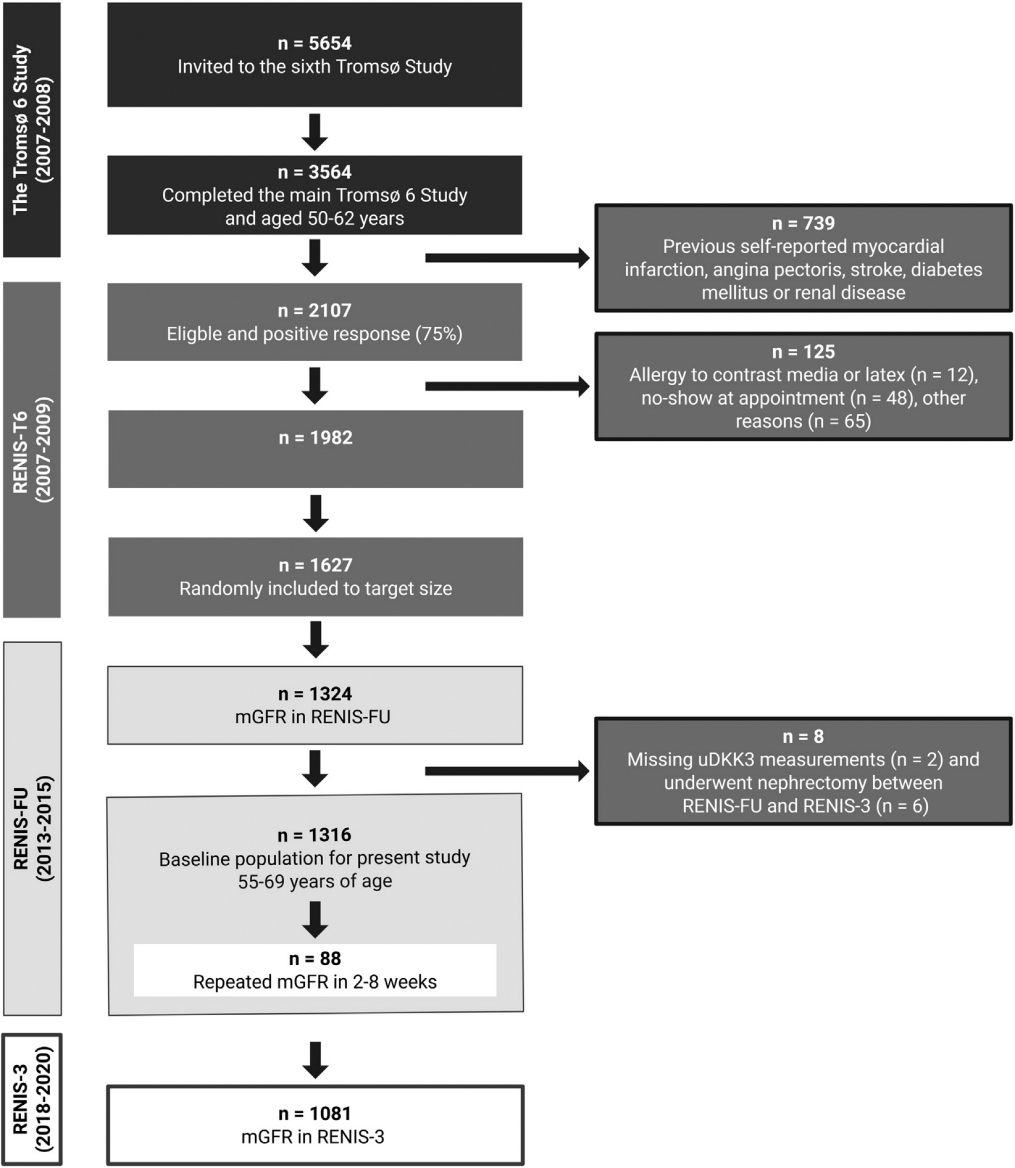
Flowchart of the RENIS cohort. mGFR, measured glomerular filtration rate; REINS-3, Renal Iohexol Clearance Survey-3; RENIS-FU, RENIS Follow-Up; uDKK3, urinary Dickkopf-3. Created in BioRender. Norvik, JV. (2025) https://BioRender.com/3vg0k9c.

Among 1627 persons investigated in RENIS-T6, 1324 participants (83%) returned for GFR measurements in the RENIS Follow-Up (RENIS-FU), conducted between 2013 and 2015, and 1174 participants (72%) had their GFR measured during RENIS-3, which spanned from 2018 to 2020. To assess the intraindividual variability of GFR, a randomly selected cohort of 88 participants from the RENIS-FU underwent a second GFR assessment. These additional measurements were incorporated into the analyses. Details about inclusion in RENIS-FU and RENIS-3 have been published previously.^15^ Because of a scarcity of urinary sample material from RENIS-T6, baseline data for the present study is from RENIS-FU, where the subjects were aged 55 to 69 years. Of the 1324 participants who attended the RENIS-FU, 2 were excluded because of missing uDKK3 measurements, and 6 were excluded for having undergone nephrectomy between the baseline and follow-up assessments. Consequently, the study population comprised 1316 subjects with a median follow-up time of 5.3 years, as detailed in Figure 1.

Ethical clearance for the research protocol was granted by the Norwegian Data Inspectorate and the Regional Committee for Medical and Health Research Ethics in Northern Norway (reference number 2016/2320/REK nord). Before participation, all subjects provided informed written consent.

### Data Collection

All participant evaluations in the RENIS-FU were consistently scheduled from 8 to 10 am, at the Clinical Research Unit of the University Hospital of North Norway. During these sessions, height and weight were recorded to determine each subject’s body mass index. BP was measured using an automated device (model UA-799; A&D), with 3 readings taken at 1-minute intervals to ensure accuracy. Participants were asked to fill out a survey detailing their medication intake and tobacco use. Use of antihypertensive medication (the use of angiotensin-converting enzyme inhibitors, angiotensin receptor blockers, beta blockers, calcium channel blockers, mineralocorticoid receptor antagonists, loop diuretics, thiazide diuretics, other diuretics, or other antihypertensive medication) was defined as yes or no; and current smoking was defined as yes or no.

Participants were instructed to fast without restricting fluid intake prior to the collection of serum samples, which were then processed for routine laboratory analyses. In addition, subjects were asked to provide first-void urine samples over 3 consecutive days during each visit. These samples were analyzed promptly to determine urinary albumin and Cr levels, from which the ACR was calculated. In the analyses, the median ACR value from the collected samples was used.

Diabetes mellitus at baseline was defined as self-reported diabetes, use of antidiabetic medication, glycated hemoglobin of 348 mmol/mol, or fasting glucose of 37 mmol/l. Metabolic syndrome was defined according to the National Cholesterol Education Program Adult Treatment Panel III 2005 criteria.

### Assessment of GFR

GFR assessment was conducted using the single-sample plasma iohexol clearance method during each visit, as described in full in a previous publication.^14^ A concise overview is as follows: an injection of 5 ml of iohexol (concentration of 300 mg/ml) was administered i.v. via a Teflon catheter placed in the antecubital vein. The timing for serum iohexol measurement was individually determined based on each participant’s eGFR, and GFR values were subsequently calculated using the formulas developed by Jacobsson. The intraindividual variability, or day-to-day coefficient of variation, for GFR measurements was 4.2%.

### Assessment of Urinary DKK3

uDKK3 levels were measured in the first void morning spot urine sample (stored at −80 °C) and quantified using a method previously detailed employing a commercially available enzyme-linked immunosorbent assay kit (DiaRen, Homburg, Germany).^8^ The interassay variability was 4.7% in the lower detection range and 5.1% in the higher detection range, indicating reliable reproducibility. The assay exhibits no cross-reactivity with other dickkopf proteins, ensuring specificity. The assay had a lower detection limit of 30 pg/ml. To adjust for urine concentration, uDKK3 concentrations were normalized to urinary Cr concentrations (uDKK3/Cr).

### Statistical Methods

GFR measurement in RENIS-FU was employed as the baseline for the analyses of measured GFR decline in this study. We stratified the cohort into 3 groups based on baseline uDKK3/Cr levels as follows: undetectable, low (detectable < 400 pg/mg), and high (≥ 400 pg/mg). An inflection point for low or high uDKK3/Cr levels is not well-defined. Another study used uDKK3/Cr levels of 400 pg/mg for low versus high uDKK3/Cr^16^ and 1 study found that subjects with uDKK3/Cr levels > 471 pg/mg had higher risk for AKI following elective cardiac surgery.^17^

Continuous variables are reported as means with SDs for normally distributed data, and as medians with interquartile ranges for data exhibiting skewed distributions. Categorical variables are expressed as percentages. To assess differences in baseline characteristics among the 3 groups categorized by uDKK3 levels, we used 1-way analysis of variance for continuous variables that followed a normal distribution. For continuous variables with skewed distributions, the Kruskal-Wallis test was used. Differences in categorical variables across the groups were evaluated using Pearson’s chi-square test. We used linear mixed models with random intercepts and random slopes, with observation time as the sole independent variable, to evaluate the annual change in GFR. To identify statistical differences in mean annual GFR change between groups in the table of baseline characteristics, we used the *P*-value derived from the 2-way interaction between observation time and the uDKK3/Cr level group.

We used linear mixed models with random intercepts and random slopes to assess the association between baseline uDKK3/Cr level group and mean annual GFR change over a median 5.3 years of follow-up.^18^

We defined accelerated GFR decline as occurring in subjects within the top 10% of the steepest GFR slopes.^18^, ^19^, ^20^ The GFR slope for each subject was calculated using all available GFR data during baseline and follow-up in a linear mixed model. This model included the baseline covariates age, sex (defined as the biological differences between males and females), body mass index, systolic BP, use of antihypertensive medication (yes/no), fasting glucose, and current smoking (yes/no) with random intercepts and slopes to account for individual variations. We then used logistic regression models to assess the association between uDKK3/Cr level group and the outcome, accelerated GFR decline.

In the linear mixed models analyses we adjusted for the following covariates: model 1: age and sex; model 2: model 1 plus office systolic BP, the use of antihypertensive medication (yes/no), body mass index, serum triglycerides, and fasting glucose; and model 3: model 2 + ACR. In the logistic regression models, baseline GFR was included in model 2 and 3. We repeated the linear mixed models, treating antihypertensive medication use as a time-varying variable. This approach allowed us to account for the impact of initiating new antihypertensive medications between baseline and follow-up on GFR decline.

We tested for effect modification by sex by including a 3-way cross-product with the uDKK3/Cr level group variable and the time variable in the linear mixed models and by including a 2-way cross-product with the uDKK3/Cr level group variable in the logistic regression analyses.

To evaluate the linear trend between uDKK3/Cr level groups and both the mean annual GFR change and accelerated GFR decline, we performed additional analyses where we treated the categorical uDKK3/Cr level group variable as a continuous variable. To explore potential nonlinear associations between the uDKK3/Cr level group and these outcomes, we included a quadratic term for the uDKK3/Cr level group in both the linear mixed models and logistic regression models. We used likelihood-ratio test to determine whether including the quadratic term improved the model fit.

To assess the discriminatory power of the urinary uDKK3/Cr in predicting an accelerated decline in GFR (defined as the 10% of individuals with the steepest decline in GFR during follow-up), we compared the area under the receiver operating characteristic curve (area under the curve) for nested logistic regression models, including the 4-variable kidney failure risk equation.^21^ The likelihood ratio test was employed to determine whether the inclusion of uDKK3/Cr level categories significantly enhanced the model's predictive capability.

All analyses were performed with the Stata 18.0 package (StataCorp, 2023. Stata Statistical Software: Release 18. College Station, TX).

## Results

The baseline characteristics of study participants are shown in Table 1, categorized by uDKK3/Cr levels: undetectable (*n* = 1112), low (detectable < 400 pg/mg, *n* = 167), and high (≥ 400 pg/mg, *n* = 37). Most characteristics were similar across groups. More women had undetectable uDKK3/Cr levels than men. Participants with high uDKK3/Cr levels had higher systolic and diastolic BPs, despite similar antihypertensive medication use. Although not statistically significant, more subjects in this group had metabolic syndrome. Baseline GFR was comparable; however, crude annual GFR decline was steeper in groups with detectable uDKK3/Cr levels. The mean GFR decline rate for the cohort was −1.33 ml/min per 1.73 m^2^. Baseline and follow-up GFR for each uDKK3/Cr level group are presented in Supplementary Figure S1.

**Table 1:** Baseline characteristics stratified by uDKK3/Cr levels into 3 categories; the subjects with undetectable uDKK3/Cr levels, the subjects with low uDKK3/Cr levels (detectable < 400 pg/mg), and the subjects with high uDKK3/Cr levels (≥ 400 pg/mg)

Characteristics	RENIS (*N* = 1316)			*P*-value
	uDKK3/Cr undetectable (*n* = 1112)	uDKK3/Cr detectable < 400 pg/mg (*n* = 167)	uDKK3/Cr ≥400 pg/mg (*n* = 37)	
Age, yrs	63.6 (4.0)	63.8 (4.0)	64.0 (3.9)	0.62
Sex, women (%)	602 (54%)	52 (31%)	10 (27%)	< 0.001
Height, cm	170.1 (8.7)	173.2 (8.1)	173.8 (7.1)	< 0.001
Weight, kg	79.3 (13.9)	80.9 (14.5)	82.9 (14.5)	0.16
Body mass index, kg/m^2^	27.3 (4.1)	26.7 (3.9)	27.4 (4.0)	0.21
Systolic blood pressure, mm Hg	130.2 (16.8)	131.8 (17.9)	139.2 (17.5)	0.004
Diastolic blood pressure, mm Hg	81.6 (9.2)	83.0 (9.8)	86.9 (9.7)	0.001
Pulse, beats/min	64.6 (9.3)	65.0 (9.5)	64.1 (10.0)	0.81
Antihypertensive medication, *n* (%)	351 (32%)	51 (32%)	13 (35%)	0.88
Current smoker, *n* (%)	140 (13%)	24 (19%)	6 (16%)	0.10
Regular use of NSAIDs, *n* (%)	67 (6%)	9 (5%)	1 (3%)	0.67
Blood glucose, mmol/l	5.5 (0.6)	5.5. (0.3)	5.6 (0.7)	0.40
HbA1c, %	5.6 (0.4)	5.6 (0.3)	5.6 (0.3)	0.61
Diabetes mellitus, *n* (%)	45 (4%)	6 (4%)	3 (8%)	0.44
LDL cholesterol, mmol/l	3.6 (0.9)	3.4 (0.9)	3.5 (1.0)	0.07
HDL cholesterol, mmol/l	1.6 (0.5)	1.6 (0.4)	1.5 (0.4)	0.08
Triglycerides, mmol/l	1.0 (0.8–1.4)	0.9 (0.7–1.2)	1.1 (0.8–1.8)	0.10
Lipid-lowering medication, *n* (%)	195 (17%)	23 (17%)	11 (30%)	0.13
Cardiovascular disease, *n* (%)	51 (4%)	10 (8%)	3 (8%)	0.19
Metabolic syndrome, *n* (%)	329 (30%)	42 (25%)	16 (43%)	0.12
Urinary albumin-to-creatinine ratio, mg/mmol	0.34 (0.10 to 0.58)	0.33 (0.10–0.62)	0.36 (0.24–0.57)	0.54
GFR, ml/min per 1.73 m^2^	89.0 [14.4]	89.6 (14.8)	89.2 (15.6)	0.89
Annual GFR change, ml/min per 1.73 m^2^/yr	−1.27 (0.43)	−1.58 (1.13)	−2.22 (2.51)	0.002
Urinary uDKK3/Cr, pg/mg	-	100.9 (37.6–220.7)	763.3 (522.5–1255.1)	-

Cr, creatinine; GFR, glomerular filtration rate; HbA1c, glycated hemoglobin; HDL, high-density lipoprotein; LDL, low-density lipoprotein; NSAIDs, nonsteroidal antiinflammatory drugs; RENIS, Renal Iohexol Clearance Survey; uDKK3/Cr, urinary Dickkopf-3/Cr.

In linear mixed models, presented in Table 2, the group with high uDKK3/Cr levels exhibited a statistically significant greater annual GFR decline than the group with undetectable levels. Over the 5.3 years of follow-up, and within the fully adjusted model, the high uDKK3/Cr level group experienced an annual GFR reduction that was 0.63 ml/min per 1.73 m^2^ more pronounced than that observed in the group with undetectable uDKK3/Cr levels. A statistically significant linear trend was observed in the association between uDKK3/Cr level groups and the mean annual change in GFR. A total of 124 subjects who were not on antihypertensive medication at baseline had initiated such treatment by follow-up. Notably, all these individuals had started either an angiotensin-converting enzyme inhibitor or an angiotensin receptor blocker during the interval between baseline and follow-up. The results from the linear mixed models, in which antihypertensive medication use was treated as a time-varying variable—thereby accounting for changes in medication use between baseline and follow-up—are presented in Supplementary Table S1. These results did not differ significantly from those presented in Table 2.

**Table 2:** Mixed linear regression analyses showing the association of uDKK3/Cr with rates of annual GFR change during a median follow-up time of 5.3 years in those with low uDKK3/Cr levels (detectable < 400 pg/mg) and those with high uDKK3/Cr levels (≥ 400 pg/mg), compared with those with undetectable uDKK3/Cr levels

Analysis	RENIS (*n* = 1316)			
	ml/min per 1.73 m^2^ per year^a^	95% CI	*P*-value	*P*-value for trend
Model 1				0.03
uDKK3/Cr undetectable (*n* = 1112)	Ref.			
uDKK3/Cr detectable < 400 pg/mg (*n* = 167)	−0.19	(−0.50 to 0.12)	0.22	
uDKK3/Cr ≥ 400 pg/mg (*n* = 37)	−0.69	(−1.31 to −0.05)	0.04	
Model 2				0.03
uDKK3/Cr undetectable (*n* = 1112)	Ref.			
uDKK3/Cr detectable < 400 pg/mg (*n* = 167)	−0.19	(−0.50 to 0.11)	0.22	
uDKK3/Cr ≥ 400 pg/mg (*n* = 37)	−0.65	(−1.28 to −0.02)	0.04	
Model 3				0.04
uDKK3/Cr undetectable (*n* =1112)	Ref.			
uDKK3/Cr detectable < 400 pg/mg (*n* = 167)	−0.18	(−0.48 to 0.13)	0.25	
uDKK3/Cr ≥ 400 pg/mg (*n* = 37)	−0.63	(−1.25 to 0.00)	0.049	

Cr, creatinine; GFR, glomerular filtration rate; uDKK3, urinary Dickkopf-3.

Model 1: adjusted for age and sex. Model 2: Model 1 plus adjusted for body mass index, office systolic blood pressure, the use of antihypertensive medications, serum triglycerides and fasting glucose at baseline. Model 3: Model 2 plus adjusted for urinary albumin-to-creatinine-ratio.

a A negative coefficient signifies a steeper decline.

**Figure 2:**
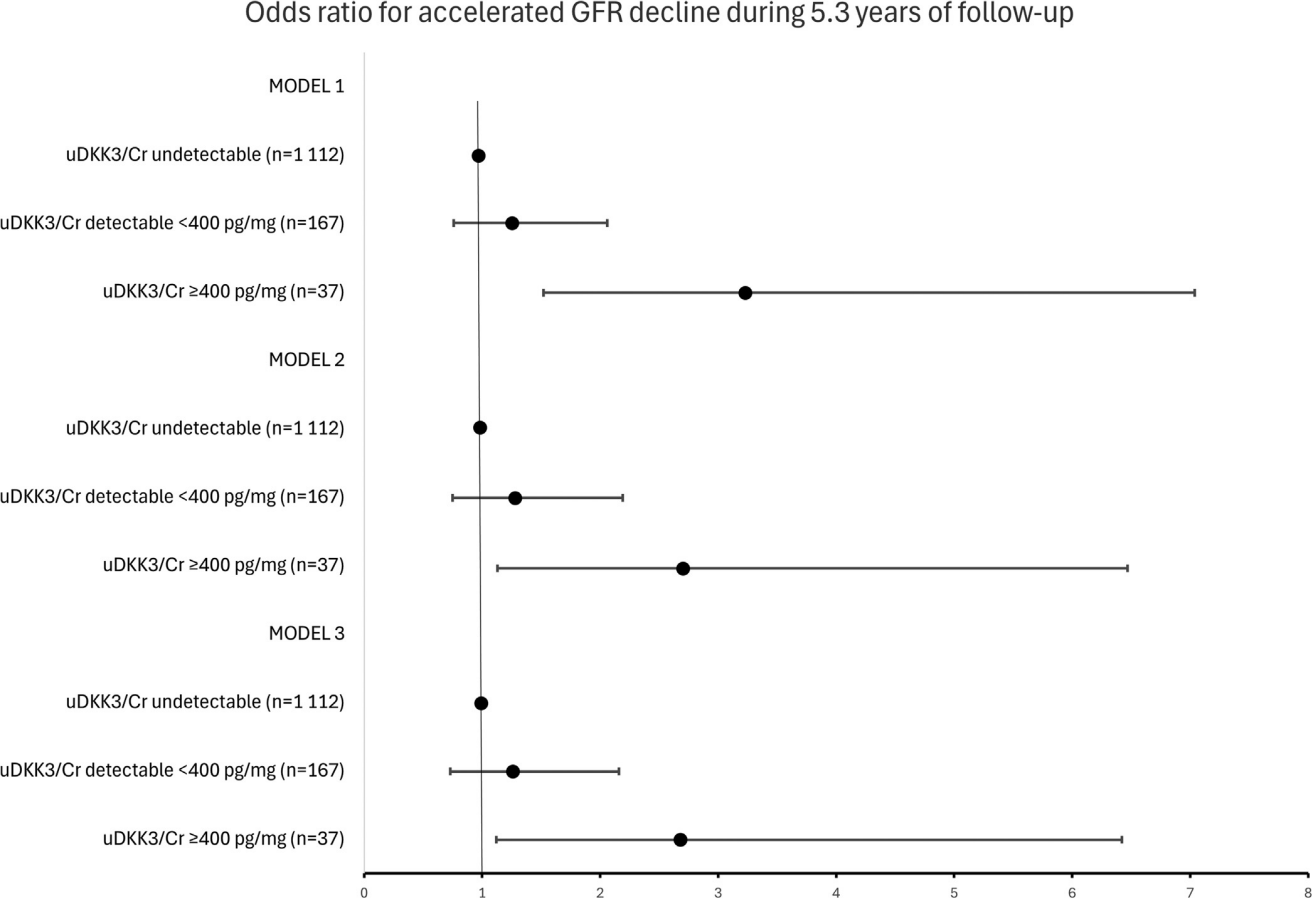
Logistic regression analyses with odds ratio for accelerated GFR decline during 5.3 years of follow-up. Model 1: adjusted for age and sex. Model 2: Model 1 plus adjusted for body mass index, office systolic blood pressure, the use of antihypertensive medications, serum triglycerides, fasting glucose and GFR at baseline. Model 3: Model 2 plus adjusted for urinary albumin-to-creatinine-ratio. GFR, glomerular filtration rate; uDKK3, urinary Dickkopf-3.

In Figure 2, we show the logistic regression analyses for accelerated GFR decline. The group with high uDKK3/Cr levels had a statistically significant higher odds ratio for experiencing accelerated GFR decline (defined as being among the 10% with the steepest decline in GFR, at 1.98 ml/min per 1.73 m^2^/yr or more) compared with the group with undetectable levels, during 5.3 years of follow-up. In model 1, adjusted for sex and age, the high uDKK3/Cr level group had an odds ratio of 3.23 for accelerated decline (*P* = 0.003). In model 3, the fully adjusted model, this group had an odds ratio of 2.68 for accelerated decline compared with the undetectable uDKK3/Cr group (*P* = 0.03). We observed a statistically significant linear trend in the association between uDKK3/Cr level groups and the outcome of accelerated GFR decline.

We evaluated the incremental value of incorporating uDKK3/Cr level categories into a statistical model for predicting accelerated GFR decline (Table 3). When uDKK3/Cr level categories were added to a model already containing the traditional CKD biomarkers GFR and ACR, the C-statistic improved from 0.59 to 0.63. When added to the 4-variable kidney failure risk equation, the C-statistic increased from 0.75 to 0.76.

**Table 3:** Change in C-statistics when adding uDKK3/Cr level category undetectable, low (detectable < 400 pg/mg), or high (uDKK3/Cr ≥ 400 pg/mg) to predict risk for accelerated GFR decline during a median 5.3 years of follow-up

Analysis	RENIS (*n* = 1316)			
	Model 1	*P*-value	Model 2	*P*-value
C-statistics without uDKK3/Cr level category (95% CI)	0.59 (0.54–0.64)		0.75 (0.71–0.78)	
C-statistics with uDKK3/Cr level category (95% CI)	0.63 (0.67–0.68)	< 0.001^a^	0.76 (0.72–0.79)	0.001^a^

Cr, creatinine; uDKK3/Cr, GFR, glomerular filtration rate; Urinary Dickkopf-3/Cr.

Model 1: adjusted for baseline GFR and albumin-to-creatinine ratio (ACR). Model 2: Model 1 plus adjusted for sex and age.

a Likelihood ratio test for model fit. Accelerated GFR decline is defined as an annual GFR decline rate of > 1.98 ml/min per 1.73 m^2^ (*n* = 131).

We found no interaction between the uDKK3/Cr level groups and sex, and we did not observe any nonlinear trends in the analyses.

## Discussion

In this prospective cohort study involving a general population who has undergone repeated GFR measurements using iohexol clearance, elevated levels of the kidney tubule-specific biomarker uDKK3 were found to be statistically significantly associated with a more rapid decline in GFR over a follow-up period of 5.3 years. Kidney tubules are integral to various physiological processes, including solute transport, acid-base homeostasis, salt-water balance, and the elimination of toxins. The established biomarkers of CKD, eGFR, and ACR, predominantly reflect glomerular function and integrity and are not sensitive to early injury or dysfunction of the kidney tubules.^6^^,^^7^ This limitation serves as a gap in the early detection capabilities of current diagnostic practices for CKD because tubular injury and dysfunction are increasingly recognized as significant aspects of CKD development and progression.^6^^,^^22^, ^23^, ^24^ Although uDKK3 is not a direct measure of kidney tubular function, it may reflect kidney tubular health indirectly by indicating stress or injury in the tubulointerstitial compartment.

uDKK3 has been examined as a biomarker of AKI across diverse cohorts. In a study of subjects with established CKD, a baseline uDKK3/Cr level > 491 pg/mg was the best threshold for identifying subjects at risk for contrast associated AKI and a baseline uDKK3/Cr level > 322 pg/mg was most effective for identifying subjects at risk for persistent kidney dysfunction 1 month from contrast administration.^10^ Another study focusing on patients undergoing coronary angiography found that uDKK3/Cr could predict contrast-associated AKI even in individuals without overt CKD.^25^ In a study of 733 subjects undergoing elective cardiac surgery, it was found that preoperative levels of uDKK3/Cr > 471 pg/mg were associated with increased risk postoperative AKI.^17^

In a cohort of subjects with established CKD from various etiologies with a mean eGFR of 48.6 ml/min per 1.73 m^2^ and a median uDKK3/Cr of 431 pg/mg, a statistically significant steeper 12-month eGFR decline was observed in subjects with uDKK3/Cr > 200 pg/mg, with the largest effect observed in subjects with uDKK3/Cr > 4000 pg/mg.^8^ The association between uDKK3/Cr levels and eGFR loss was validated in another CKD cohort, the STOP IgA Nephropathy Trial, in subjects with IgA nephropathy.^8^ A study of 2 CKD cohorts, which reported median levels of uDKK3/Cr of 2200 and 3042 pg/mg, found that uDKK3/Cr > 5075 pg/mg was associated with CKD progression.^9^ The uDKK3 levels reported in CKD cohorts illustrate the contrast in uDKK3 levels between patients with CKD and the general population. In the current study, uDKK3/Cr levels were undetectable in 84% of participants, perhaps posing a challenge for its use as a widespread diagnostic tool. However, uDKK3/Cr may serve as a valuable biomarker for identifying individuals with healthy kidney tubules, indicating a lower risk of experiencing a more rapid decline in kidney function.

In children, uDKK3/Cr has been demonstrated to predict short-term eGFR decline. In an observational cohort study of 659 children with CKD of various etiologies, participants with uDKK3/Cr levels above the median (> 1689 pg/mg) had a significantly steeper 6-month eGFR decline than those with uDKK3/Cr levels below the median, independent of eGFR and albuminuria.^26^ Similarly, in a study of 195 pediatric patients with renal ciliopathies, and where a minority had proteinuria and hypertension, uDKK3/Cr levels were found to correlate with CKD severity, and uDKK3/Cr values > 4700 pg/mg were associated with a steeper annual eGFR decline.^27^

Although not within a general population cohort, uDKK3 has been evaluated as a biomarker for longer-term loss of kidney function in a cohort without established CKD. Specifically, in a study involving 2314 patients with chronic obstructive pulmonary disease, baseline levels of uDKK3/Cr effectively identified patients at high risk for significant eGFR decline during the follow-up period.^28^ In contrast, traditional markers such as eGFR and proteinuria did not distinguish these at-risk patients. In a separate study, uDKK3/Cr levels were found to be predictive of eGFR decline in patients with heart failure during a median of 13 months. Notably, 69% of the study cohort did not have CKD, as evidenced by an eGFR > 60 ml/min per 1.73 m^2^ and albuminuria < 30 mg/g.^29^

Several tubular biomarkers associated with kidney outcomes have been identified in the recent years.^20^^,^^23^^,^^24^^,^^30^ In the present study, uDKK3/Cr demonstrated performance that was comparable to or exceeded that of many other proposed CKD biomarkers.^31^^,^^32^ In a previous study from our cohort, we measured the tubular biomarkers kidney injury molecule-1, uromodulin, and monocyte chemoattractant protein-1 in serum samples; however, none of these markers were found to be statistically significantly associated with GFR decline.^33^ In another study from our cohort, we examined the tubular biomarker urinary epidermal growth factor in relation to GFR decline, and uDKK3 improved the C-statistic to a comparable extent as urinary epidermal growth factor.^20^ An increase in C-statistics of 1%, however, may not be a clinically significant enhancement on the 4-variable kidney failure risk equation. Because individual biomarkers may not consistently enhance existing risk prediction methods to a clinically significant extent, Ix and Shlipak^6^ proposed the adoption of a “kidney test panel” that collectively will improve prediction, similar to a liver test panel, as a future standard in clinical practice. This panel would not only include markers of glomerular health, such as eGFR and ACR, but also biomarkers indicative of tubular dysfunction and injury. Our validation of uDKK3 as a tubular biomarker of GFR loss in this general population cohort, assessed by measured GFR, supports the inclusion of uDKK3 in such a comprehensive kidney health biomarker panel.

The strengths of our study lie in the repeated GFR measurements conducted within a thoroughly characterized cohort that is representative of the general population. In addition, the study benefited from a high participation rate and demonstrated low day-to-day variation in GFR measurements, enhancing the reliability of the findings. However, the study cohort consisted almost exclusively of individuals of Northern European ancestry, which limits the generalizability of the findings to more diverse populations. Furthermore, the cohort did not include any participants aged < 55 years, which limits the generalizability of the findings to younger populations. Another potential limitation is that the majority of the cohort had undetectable uDKK3/Cr levels. Consequently, the observed association between uDKK3/Cr values > 400 pg/mg and a more rapid GFR decline was derived from a relatively small subset of 37 individuals, which may restrict the generalizability of our findings.

In conclusion, higher levels of uDKK3 were associated with a steeper decline in measured GFR, independent of baseline GFR and ACR, over a follow-up period of 5.3 years. This study has validated findings from other research—primarily conducted in CKD cohorts using eGFR—in this general population cohort assessed by measured GFR via iohexol clearance. These results suggest that uDKK3 could be a valuable biomarker in a panel designed for early detection of GFR loss, enhancing intervention strategies for kidney health preservation.

## Disclosure

DF is affiliated with DiaRen UG. All the other authors declared no competing interests.

## Supplementary Material

Supplementary File (PDF) **Figure S1.** Baseline and follow-up GFR in ml/min per 1.73 m^2^ for each uDKK3/Cr level group: undetectable (*n* = 1112), low (detectable < 400 pg/mg, *n* = 167), and high (≥ 400 pg/mg, *n* = 37). **Table S1.** Mixed linear regression analyses showing the association of uDKK3/Cr with rates of annual GFR change during a median follow-up time of 5.3 years in those with low uDKK3/Cr levels (detectable < 400 pg/mg) and those with high uDKK3/Cr levels (≥ 400 pg/mg), compared with those with undetectable uDKK3/Cr levels, with the use of antihypertensive medication as a time-varying variable. STROBE Checklist.
